# A jumping crystal predicted with molecular dynamics and analysed with TLS refinement against powder diffraction data

**DOI:** 10.1107/S205225251801686X

**Published:** 2019-01-01

**Authors:** Jacco van de Streek, Edith Alig, Simon Parsons, Liana Vella-Zarb

**Affiliations:** a Department of Pharmacy, University of Copenhagen, Copenhagen, Denmark; b Institute for Inorganic and Analytical Chemistry, Goethe-University Frankfurt, Frankfurt am Main, Germany; c School of Chemistry/Centre for Science at Extreme Conditions, University of Edinburgh, Edinburgh, UK; d Chemistry Department, University of Malta, Msida, Malta

**Keywords:** jumping crystals, molecular dynamics, phase transitions, TLS refinement, XRPD

## Abstract

By running a temperature series of molecular dynamics (MD) simulations starting from the known low-temperature phase, the experimentally observed phase transition in a ‘jumping crystal’ was captured, thereby providing a prediction of the unknown crystal structure of the high-temperature phase and clarifying the phase-transition mechanism. The phase transition is accompanied by a discontinuity in two of the unit-cell parameters. The structure of the high-temperature phase is very similar to that of the low-temperature phase. The anisotropic displacement parameters calculated from the MD simulations readily identified libration as the driving force behind the phase transition. Both the predicted crystal structure and the phase-transition mechanism were verified experimentally using TLS (translation, libration, screw) refinement against X-ray powder diffraction data.

## Abstract 2

A phase transition in a jumping crystal is predicted using molecular dynamics. The resulting structure and the dynamics of the transition are verfied by TLS refinement against powder diffraction data.

## 1. Introduction   

Occasionally, interesting crystal structures can be prepared by applying temperature or pressure to a crystalline phase in order to trigger a phase transition and obtain a different solid phase. Examples are the preparation of co-crystals by solid-state grinding (Trask & Jones, 2005 ▸), the dehydration of hydrates to prepare anhydrates (Fujii *et al.*, 2012 ▸) or the heating of crystals to make them ‘jump’ (Sahoo *et al.*, 2013 ▸). These cases have in common that the structures of the starting phases and the preparation processes are known, but the structures of the end products are difficult to determine because the crystals tend to shatter in the process, leaving nothing but a sample in powder form to work with. Indeed, in these cases the crystal structure of the resulting phase, which is often the phase of interest, must be determined from powder diffraction data (Trask *et al.*, 2005 ▸; Bond *et al.*, 2014 ▸; Panda *et al.*, 2014 ▸).

This led us to consider a computational approach to the determination of the structure of the new phase based on the two pieces of information that are known: the structure of the starting phase and the preparation process of the new phase. The known starting structure can be recreated computationally by means of molecular modelling (or, in the ideal case, by crystal structure prediction), whereas the process can be recreated computationally by means of molecular dynamics (MD). In other words, when the known (or predicted) starting structure is heated *in silico* with MD, we expect to observe a phase transition, the product of which hopefully corresponds to the phase of interest.

As a model system, we used a jumping crystal for which the crystal structure of the initial phase is known, whereas that of the post-salient phase is not: *trans*,*trans*,*anti*,*trans*,*trans*-perhydro­pyrene (**I**). The crystal structure of the initial phase (*P*2_1_/*c*, *Z*′ = 2 × ½) was reported in 1991, and the crystals are reported to jump and shatter at 344.5 K (Ding *et al.*, 1991 ▸).

The success of our approach was subsequently verified by temperature-dependent powder diffraction experiments. A TLS (translation, libration, screw) refinement of the experimental data confirmed the phase-transition mechanism observed in the MD simulations.[Chem scheme1] 
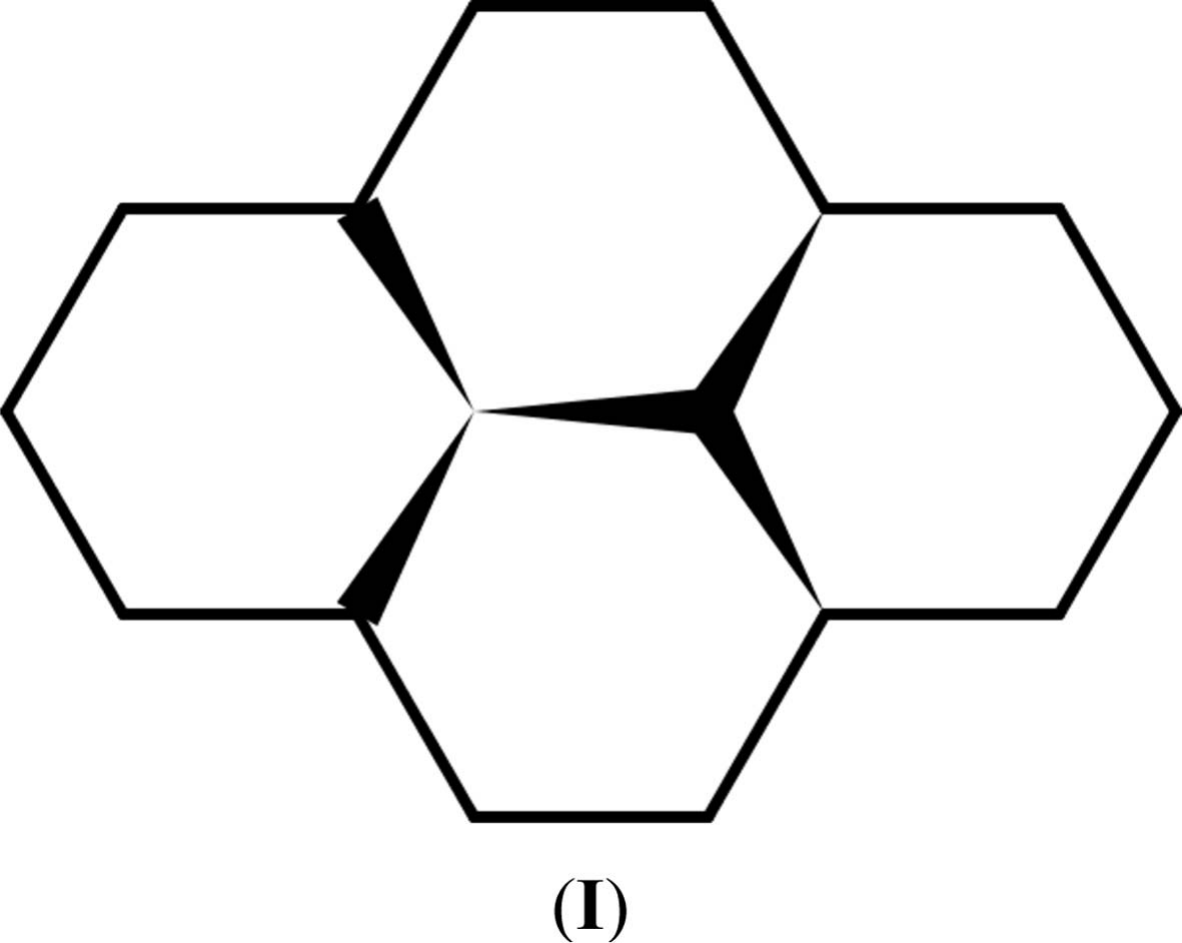


## 2. Methods   

### 2.1. Molecular dynamics   

**Figure 1:**
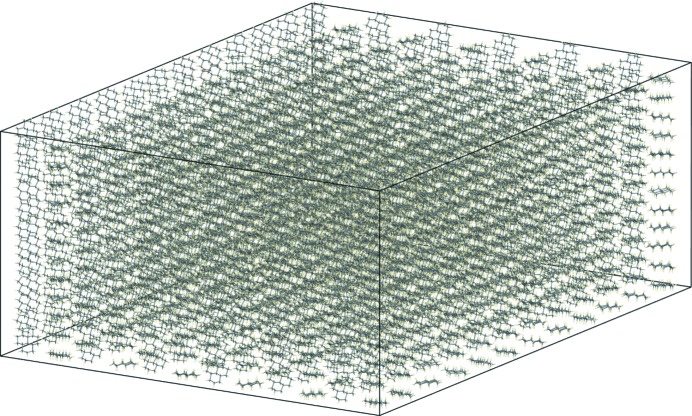
The simulation box containing 12 × 12 × 12 = 1728 independent molecules as used in the MD simulations. A snapshot at 275 K is shown.

The crystal structure of the low-temperature phase was taken from the Cambridge Structural Database (Groom *et al.*, 2016 ▸; reference code KOFHOC). To ensure that our calculations could have been performed even in the absence of any experimental data, to allow for the possibility of a true prediction, the experimental crystal structure was first energy minimized with an unrestrained unit cell and dispersion-corrected density functional theory (DFT-D). This is the crystal structure that would have been obtained from a modern-day crystal structure prediction study (Reilly *et al.*, 2016 ▸), even in the absence of experimental data, provided the study had been successful in locating the most experimentally stable polymorph. DFT-D is too computationally expensive to be used for MD, and the COMPASS force field (Sun, 1998 ▸) was used for classical MD simulations. The DFT-D crystal structure was energy-optimized, with an unrestrained unit cell, with the COMPASS force field to yield a structure corresponding to *T* = 0 K, the temperature of which was then slowly increased. The *Forcite Plus* module in *Materials Studio* was used, periodic boundary conditions were applied, the time step was 1 fs and the space group of all the simulation cells was *P*1. To allow the use of a relatively large cut-off distance for non-bonded interactions (electrostatic and van der Waals) and to reduce the self-interactions introduced by the periodic boundary conditions, relatively large supercells must be used. The cut-off distance for both types of non-bonded interaction was 25 Å and each side-to-side distance of the supercell was not less than 50 Å (twice the cut-off distance). Furthermore, the number of molecules in each direction of the simulation cell must be commensurate with the number of molecules in the starting structure and the unknown target structure. This can be achieved by constructing a simulation box containing 12 × 12 × 12 = 1728 molecules, because 12 is a multiple of 1, 2, 3, 4 and 6, allowing a phase transition to virtually all of the 230 possible space groups.^1^ For the low-temperature phase of **I**, the 12 × 12 × 12 simulation box had dimensions *a* = 99.46 Å, *b* = 62.95 Å, *c* = 97.86 Å, α = 90.0°, β = 119.16°, γ = 90.0° and *V* = 535 123 Å^3^ at 0 K. Fig. 1 ▸ gives an impression of the dimensions of the simulation box.

The COMPASS force field comes with its own atomic point charges, eliminating the ambiguity regarding the parameterization of the atomic charges associated with many force fields. The COMPASS force field is parameterized to be used without Ewald summation; instead, charge groups equating to whole molecules were used to ensure charge neutrality of the Coulomb summations.

To bracket the temperature of the anticipated phase transition, MD simulations were carried out at intervals of 50 K, starting at 50 K. At each temperature, the atomic velocities were initialized with the final values from the previous temperature. The first MD simulation, at 50 K, was initialized with random atomic velocities. Because our systems are extremely small compared with real-life systems (of the order of 1000 molecules) and the time scales are extremely short (several ps), the sudden increase in temperature by 50 K represents a severe temperature shock for the system, and experimenting with different equilibration protocols showed that careful equilibration is needed to avoid artefacts. The equilibration at each temperature was split into three steps. First, the NVT ensemble and the Berendsen thermostat (Berendsen *et al.*, 1984 ▸) were used; in this step the cell parameters were fixed and the simulation time was 2.0 ps. Second, the NPT ensemble and the Berendsen thermostat and barostat (allowing for isotropic scaling of the unit-cell volume only) were used for 3.0 ps. In the third step, the NPT ensemble was used for 20.0 ps, with the Nosé–Hoover–Langevin (NHL) thermostat (Samoletov *et al.*, 2007 ▸) to control the temperature and the Parrinello barostat (Parrinello & Rahman, 1981 ▸) to control the pressure. The equilibration was followed by a 25 ps NPT production run that was used for the calculation of the average unit-cell parameters. The temperature series was continued up to the melting point, which occurred in the simulation at 500 K, to exclude the possibility of a second phase transition in the MD simulations. So in total, 40 successive MD simulations were run, each simulation starting from the final positions and velocities of the previous one, with a total duration of 500 ps. At each temperature, the unit-cell parameters were sampled 432 times and averaged. The first temperature series put the phase-transition temperature between 250 and 300 K, which was refined to between 250 and 275 K with three additional MD runs at 225, 275 and 325 K.

From the 12 points of the temperature series, it is largely the phases just before and just after the phase transition that are of interest. These two phases were investigated with greater precision by first running a long (1 ns) NPT simulation from which the average unit-cell parameters were determined, followed by a long (1 ns) NVT simulation with the average unit-cell parameters from the NPT simulation imposed. The fixed unit-cell parameters in the second simulation are intended to mimic the effect of a much larger external crystal surrounding our simulation box and are necessary to yield correct anisotropic displacement parameters that are free from the effects of unit-cell fluctuations. The MD trajectory was sampled every 1 ps, *i.e*. averages were calculated over 1001 snapshots (frames). The space group of the new phase was determined from the 12 × 12 × 12 *P*1 supercell averaged over 1001 frames with the space-group perception tool in *Materials Studio* with a tolerance of 0.2 Å. Once the space group of the new phase had been established, which was determined to be *P*2_1_/*c*, *Z*′ = ½, the supercells were collapsed onto the asymmetric unit and all symmetry-equivalent atoms were averaged over space and time. In other words, for the calculated structure of the new phase, each atomic coordinate was the average of 1001 × 12 × 12 × 12 × 2 = 3 459 456 values. The unit-cell parameters of the new phase were obtained by averaging over the 1001 frames. From these two 1 ns NVT simulations, the time-averaged structures, the anisotropic displacement parameters and the X-ray powder diffraction patterns were calculated using in-house software.

On modern hardware, the calculations are relatively trivial in terms of resources. MD simulations do not require the huge amounts of memory (RAM) that are characteristic of quantum-mechanical calculations (such as DFT calculations). The algorithms have been parallelized, so by running the simulations on, 48 cores simultaneously for example, the wall-clock time of all the simulations described in the current work amounts to only a couple of weeks.

Minor rounding errors in the integration of Newton’s equations of motion lead to a minor but noticeable drift of the centre of mass of the contents of the simulation cell. This drift is nonphysical and was removed by resetting the centre of mass of all frames to that of the first frame. The values of the drift corrections were manually inspected to ensure that they were very small and not indicative of any problems in the calculations.

### 2.2. Synthesis   

A mixture of isomers of perhydro­pyrene (1 g) was purchased from Sigma–Aldrich with a purity of 95%. Purification of the mixture of isomers followed the procedure published elsewhere (Langer & Lehner, 1973 ▸). Benzene, *n*-hexane, di­chloro­methane and ethanol were obtained from Scharlau (reaction grade). AlCl_3_ was purchased from Alfa Aesar (anhydrous, 99.985% metal basis). All manipulations which included the use of dry AlCl_3_ were carried out under exclusion of air and moisture using a Schlenk-line.

A total of 1 g (4.58 mmol) of the mixture of isomers of **I** (MW 218.38 g mol^−1^) was dissolved in 20 ml of freshly distilled benzene and 670 mg of AlCl_3_ was added; the resulting solution was heated under reflux for 2 h.

The following steps were carried out in open air. After the solution was left to cool completely, 50 ml of 2 *M* HCl and 150 ml CH_2_Cl_2_ were added and stirred rapidly for 15 min. The organic phase was separated from the aqueous phase and washed three times with distilled water. Drying over NaSO_4_ and removal of the solvent resulted in a dark yellow/orange oil. In order to separate the dehydration products from **I**, column chromatography was carried out using Al_2_O_3_ (column width 3 cm, length 10 cm) with *n*-hexane as eluate. Subsequent evaporation of the collected fractions and recrystallization in absolute ethanol yielded 296.5 mg of **I** (30% of the theoretical yield). The melting point of 103 °C confirmed the correct and pure product.

### 2.3. Powder diffraction   

X-ray powder diffraction data were recorded at 350 K on a Stoe Stadi-P diffractometer equipped with a focusing Ge(111) monochromator and a linear position-sensitive detector using Cu *K*α_1_ radiation. The sample was contained in a glass capillary with 1.0 mm diameter, which was spun during the measurement. Data were collected in a 2θ range from 2 to 100° with a step width of 0.01° and a total data collection time of about 18 h. The software *WinX^POW^* (Stoe & Cie, 2009 ▸) was used for data acquisition.

### 2.4. Rietveld refinement   

Rietveld refinement was carried out with *TOPAS* (Coelho, 2018 ▸). To ensure that the Rietveld refinement was as robust and smooth as possible, it was carried out in stages, gradually releasing more and more parameters. The first two stages consisted of Pawley refinements to establish suitable starting values for the unit-cell parameters and the peak-shape functions, which were then transferred to the Rietveld refinement. In the last cycle, all parameters were refined simultaneously, a requirement for the proper evaluation of the goodness of fit, which depends on the number of parameters included in the fit. Anisotropic peak broadening and peak asymmetry were included to allow the peak profiles to be described accurately. Since in both structures the molecules are situated on inversion centres, the asymmetric units in principle contain only five of the ten carbon atoms of the two central cyclo­hexane rings. However, the five symmetry-related carbon atoms missing from the list of atoms prevent the specification of several of the bond-length restraints and valence-angle restraints. Therefore, the entire molecule was specified in the input file but with appropriate symmetry constraints to ensure that the number of degrees of freedom did not change. All restraints can then be expressed in natural variables such as C—C bond lengths and C—C—C valence angles without the need for dummy atoms.

Three Rietveld refinements with three different models for the description of the thermal parameters were carried out. The first refinement used one global isotropic thermal parameter *U*_iso_ for all non-hydrogen atoms. The hydrogen atoms were assigned an isotropic displacement parameter equal to 1.2 times the isotropic displacement parameter for the non-hydrogen atoms. This is the model that we traditionally adopt when working with laboratory powder diffraction data. The second Rietveld refinement used the anisotropic displacement parameters (ADPs) calculated from the MD simulations, scaled by a linear scale factor. In this model, all atoms, including the hydrogen atoms, are described fully anisotropically, but only a single parameter is fitted, similar to the case of using a global *U*_iso_. The third Rietveld refinement used the TLS model described in detail below.

### 2.5. TLS refinement   

It is generally not possible to refine anisotropic displacement parameters from powder diffraction data because the peak overlap reduces the information available, not leaving enough to refine six independent parameters per non-hydrogen atom. However, if it is assumed that the molecule behaves as a rigid body, then the thermal motion of the molecule as a whole can be described by means of a single set of TLS (Translation, Libration, Screw) parameters. For compound **I**, this reduces the number of parameters needed to describe the thermal motion of the non-hydrogen atoms in the harmonic approximation from 48, when individual ADPs are used, to 20 when ADPs from a rigid-body approximation are used. In addition, in both polymorphs the molecules are on an inversion centre, as a result of which the screw tensor vanishes, further reducing the number of parameters to just 12 (Schomaker & Trueblood, 1968 ▸; Dunitz *et al.*, 1988 ▸; Downs, 2000 ▸).

The specification of the TLS refinement in the *TOPAS* input file requires a conversion from fractional to Cartesian coordinates and from Cartesian coordinates to the ADPs in CIF format (Grosse-Kunstleve & Adams, 2002 ▸), requiring many lines of equations for each atom.

The DFT-D energy minimization from which the MD simulations were started provides us with near-perfect bond lengths and valence angles that can be used as restraints in the Rietveld refinement (see *e.g*. van de Streek, 2015 ▸). Librational thermal motion however, shortens the bond lengths calculated from the average atomic positions, while the DFT-D energy minimization is a *T* = 0 K calculation and therefore describes the molecule at rest. TLS refinement describes the motion of a rigid body and the TLS parameters can be used to correct the intramolecular distances of the rigid body for the effects of the motion (Downs, 2000 ▸). In other words, the TLS parameters can be used to calculate the coordinates of the underlying rigid body as if it were at rest. We can then apply the bond-length restraints and the valence-angle restraints from the DFT-D energy minimization to the bond lengths and valence angles of the static rigid body recovered from the TLS refinement. The thermal motion correction was implemented in the *TOPAS* input file and the bond-length and valence-angle restraints from the DFT-D minimization were used in the Rietveld refinement.

In total, about 2000 lines of equations were required for the TLS refinement of **I** on an inversion centre with restraints corrected for thermal motion. An example *TOPAS* input file can be found in the supporting information.

## 3. Results and discussion   

### 3.1. Molecular dynamics   

**Figure 2:**
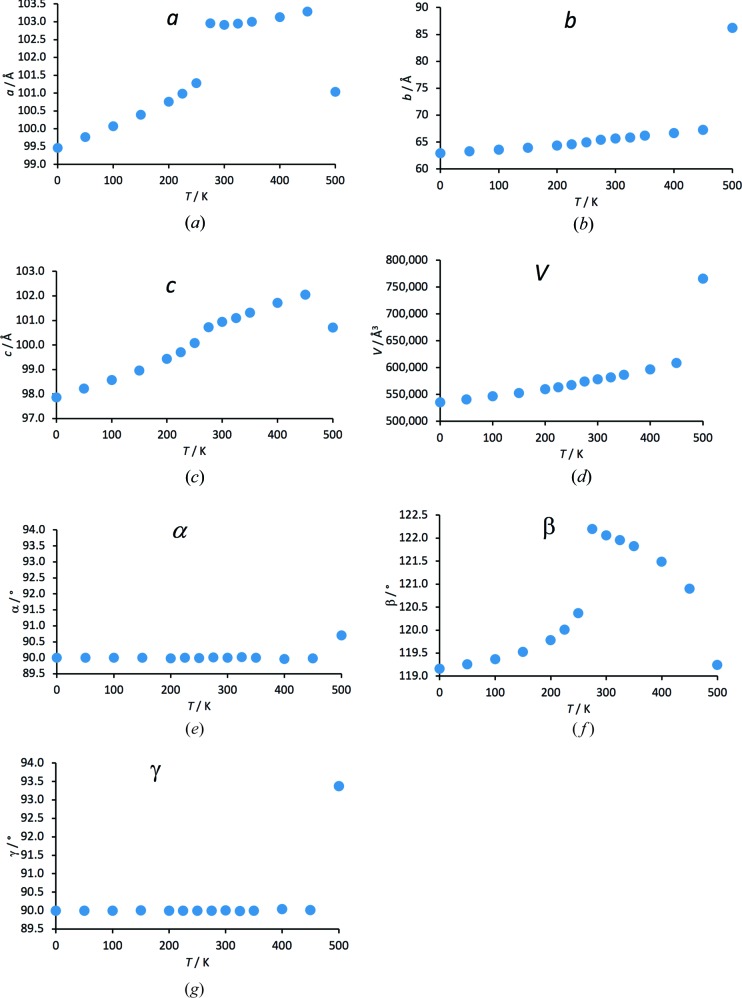
Unit-cell parameters and unit-cell volume as a function of temperature in the MD simulations. The phase transition is easily identified between 250 and 275 K. At 500 K the crystal melts.

**Figure 3:**
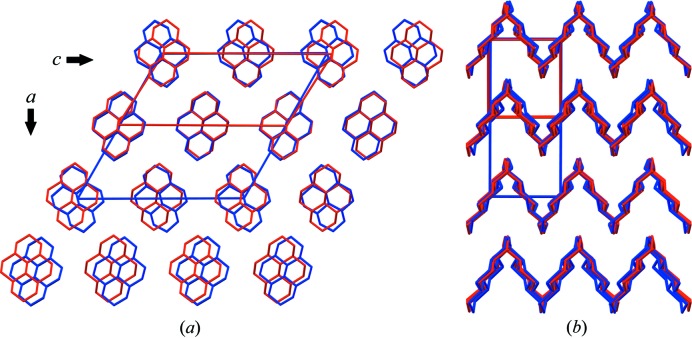
Overlay of the low-temperature phase (blue) and the high-temperature phase (red) viewed along **b** (*a*) and along **c** (*b*). These overlaid representations show that the difference between the two phases is restricted to the two directions in the plane of the molecules.

The results of the MD simulations were analysed by plotting the unit-cell parameters as a function of temperature, immediately revealing a discontinuity in the unit-cell parameters *a* and β between 250 and 275 K (Fig. 2 ▸). The *b*, *c* and unit-cell volume parameters appeared to change smoothly; α and γ remained at 90° (Fig. 2 ▸). Applying the symmetry-perception algorithm in *Materials Studio* to the time-averaged NVT structure at 275 K (after the phase transition) consisting of 12 × 12 × 12 molecules yielded a structure in *P*2_1_/*c*, *Z*′ = ½, with the molecule occupying a centre of symmetry. Indexing of the powder diffraction pattern calculated from the MD trajectory gave the same result. The low-temperature and high-temperature phases are very similar and differ only in a minor reorientation of the molecules in the plane of the molecule [Fig. 3 ▸(*a*)]; when viewed along the plane of the molecule, the two structures are virtually identical [Fig. 3 ▸(*b*)]. A similar case of a crystal jumping upon heating while undergoing a transformation from a low-temperature phase to a structurally very similar high-temperature phase due to an anisotropic expansion (shown by three unit-cell parameters changing abruptly while the other unit-cell parameters showed no pronounced discontinuity) has been investigated with single-crystal X-ray diffraction (Lusi & Bernstein, 2013 ▸). In MD simulations of phase transitions upon heating in several other molecular compounds (van de Streek *et al.*, unpublished results), all changes in the unit-cell dimensions were consistently smooth, suggesting that the jumping of crystals is associated with a discontinuity in at least one unit-cell parameter.

The new high-temperature phase is a local minimum in the DFT-D potential, *i.e*. at *T* = 0 K, with an energy that is only 0.1 kcal mol^−1^ higher than that of the low-temperature form.

**Figure 4:**
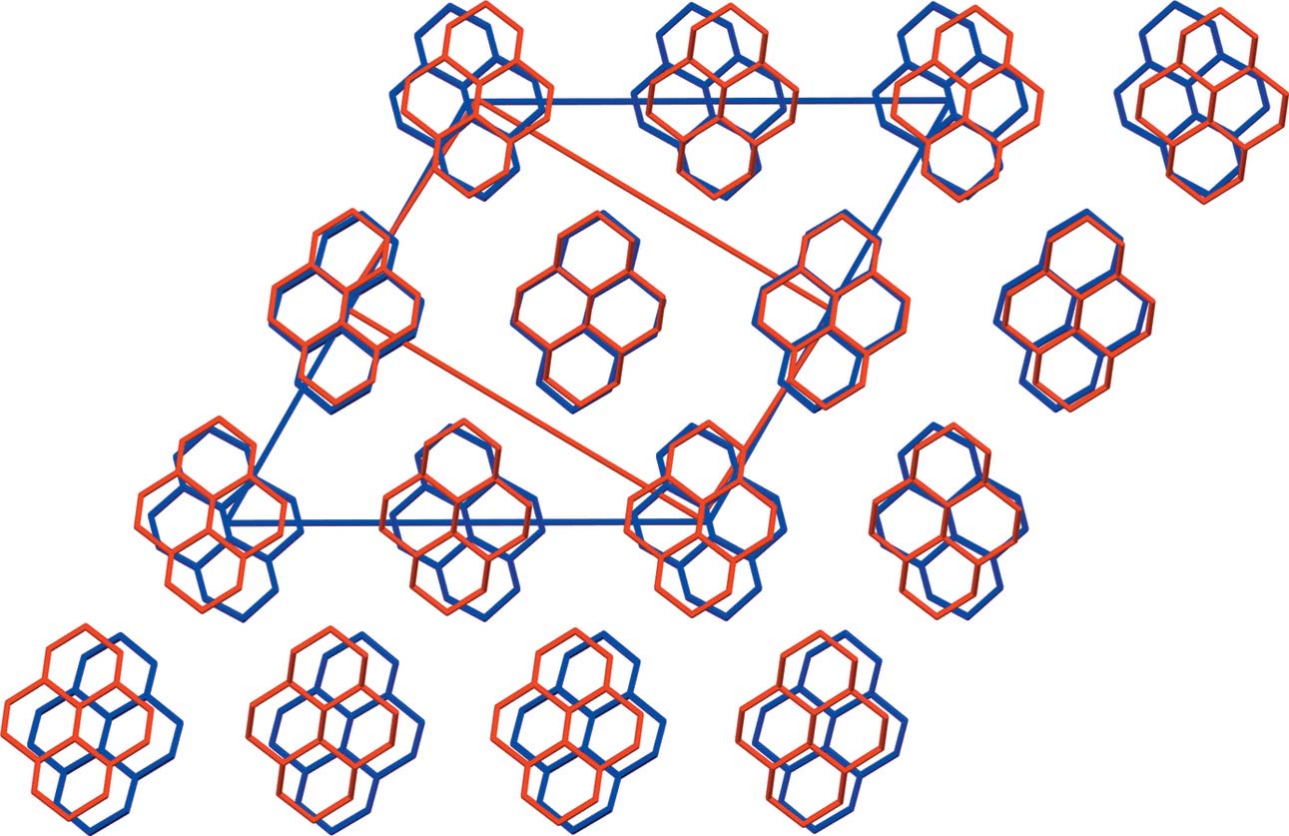
Overlay of the low-temperature phase (blue) and the alternative unit-cell setting with β = 92° (*P*2_1_/*n*, *Z*′ = ½) of the high-temperature phase (red).

The unit cell shown in Fig. 3 ▸(*a*), with β = 122°, can be transformed to a unit cell with β closer to 90° (Fig. 4 ▸, β = 92°) by applying the unit-cell transformation [101, 010, −100]. The space-group setting then becomes *P*2_1_/*n*, *Z*′ = ½.

The ADPs calculated from the MD trajectory, which represent the thermal motion of the molecules, are shown in Fig. 6(*a*). To allow a direct quantitative comparison to the ADPs fitted directly to the powder data, the ADPs from the MD simulations at 275 K were fitted against the powder diffraction data at 350 K with a linear scale factor (Blessing, 1995 ▸). The scale factor refined to a value of 1.50 (2). The ADPs beautifully illustrate the mechanism behind the phase transition: the molecules librate in the plane of the molecule until the orientations of the two symmetry-independent molecules line up and the molecules become translationally equivalent. This explanation is also in agreement with the similarity between the two phases when viewed along the plane of the molecules [Fig. 3 ▸(*a*)].

The predicted phase-transition temperature of about 260 K is about 85 K lower than the experimental transition temperature of 344 K. The computationally modelled crystals are free from defects, impurities and interfaces which, combined with the short simulation times, systematically shift the transition temperatures in MD simulations to higher temperatures, so 260 K is an upper limit. The discrepancy of more than 85 K between the experimental and the simulated transition temperature can be attributed to the inaccuracy of the energy potential used in the MD simulations. Suggestions to improve the accuracy of the force field are given below.

### 3.2. Rietveld refinement   

**Figure 5:**
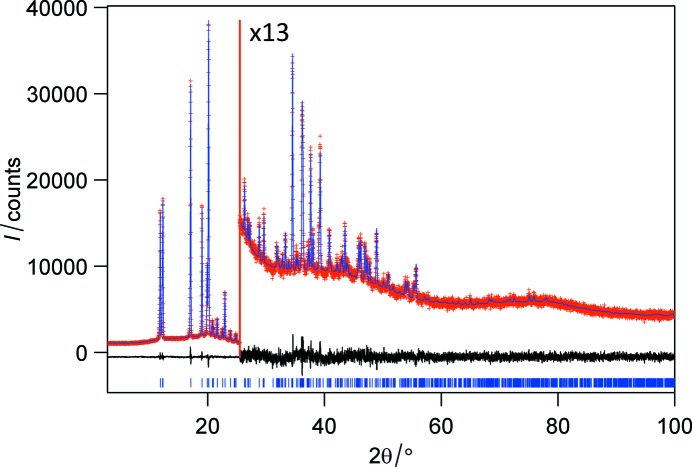
Fitting of the calculated to the experimental XRPD pattern after Rietveld refinement. Calculated (blue), observed (red) and difference (black) profiles are shown. Tick marks, indicating the calculated positions of the diffraction peaks, are shown at the bottom of the profile. The range above 25° 2θ was expanded by a factor of 13.

**Figure 6:**
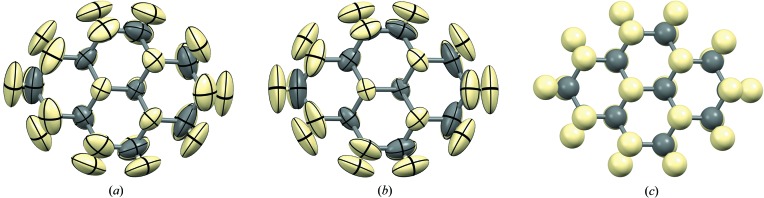
(*a*) Calculated ADPs of the high-temperature phase at 350 K (after ‘jumping’) from the MD simulations at 275 K with a scale factor fitted to the powder diffraction data at 350 K. (*b*) Experimental ADPs from the TLS refinement at 350 K. (*c*) Global isotropic *U*_iso_ fitted to the powder diffraction data at 350 K.

Fig. 5 ▸ shows the Rietveld refinement. When only one isotropic thermal parameter for all non-hydrogen atoms was refined, it refined to the value *U*_iso_ = 8.94 (7) Å^2^ [Fig. 6 ▸(*c*)]. Table 1 ▸ shows various parameters characterizing the three Rietveld refinements.

### 3.3. TLS refinement   

The ADPs obtained from the TLS refinement are shown in Fig. 6 ▸(*b*), confirming that the experimental and the simulated ADPs are very similar. The current structure must be one of very few structures determined from X-ray powder diffraction data – in this case even laboratory X-ray powder diffraction data – for which credible anisotropic displacement parameters for the hydrogen atoms were fitted directly against the X-ray data without the need for complementary experimental techniques. This is only possible of course because of the rigid-body approximation, such that the motion of the hydrogen atoms is effectively defined by the motion of the non-hydrogen atoms. This is a generally applicable method for the calculation of anisotropic displacement parameters for hydrogen atoms, and is well developed for single-crystal structures (Madsen & Hoser, 2014 ▸).

The experimental ADPs as a function of temperature can be analysed quantitatively to obtain information about the reaction pathway [Hummel, Hauser *et al.* (1990 ▸); Hummel, Raselli *et al.* (1990 ▸); see also footnote 73 in Skoko *et al.* (2010 ▸)], but this was beyond the scope of the current study.

### 3.4. Validity of the rigid-body approximation   

The TLS refinement against the XRDP data is based on the assumption that the molecule is rigid. To date, we have not presented any evidence to support this assumption other than to allow the reader to visually inspect **I** and to conclude, as we did, that this is a reasonable assumption. Indeed, in tests on a handful of other compounds for which the rigid-body approximation seemed unlikely to apply, the ADPs quickly became non-positive definite, the refinements did not converge properly and the ADPs of atoms far from the centre of the molecule refined to very extreme shapes. Dunitz *et al.* (1988 ▸) described a quantitative test to check if the thermal motion of the individual atoms supports an approximation as a rigid body: the average displacement along a vector connecting any two atoms must be identical for the two atoms. In practice, this is implemented by fitting individual ADPs for all atoms, *i.e*. without the rigid-body assumption, so that the average displacements can be calculated and compared. This approach, however, is not applicable in our case because the individual displacements are not accessible for powder diffraction data, so the test cannot be carried out.

Therefore, the most critical assessment of the reliability of the ADPs reported here is limited to the observation that two entirely independent methods (MD and TLS refinement against experimental data) give the same reasonable result, which furthermore suggests a phase-transition mechanism consistent with the similarities and differences between the two phases (Fig. 3 ▸), and that the rigid-body approximation is valid and hence our ADPs are probably correct.

### 3.5. Extension to other systems: TLS refinement   

The success of our TLS refinement hinges crucially on two factors. First, compound **I** is clearly described very well as a rigid body in the solid state. When trying to apply the TLS refinement to several other Rietveld refinements of pigments and pharmaceuticals, it quickly became clear that in the general case of a molecular compound, indiscriminately treating the whole molecule as a rigid body invariably leads to non-positive definite atoms, convergence problems in the refinement and extreme ADPs for atoms far from the centre of the molecule. In principle, this can be solved by partitioning the molecule into smaller fragments, each of which can be described as a rigid body (Painter & Merritt, 2006*a* ▸,*b* ▸). However, to start with, in the absence of individual ADPs for refinements against powder diffraction data, such a partitioning requires a substantial amount of trial and error with no quantitative measure for success available and, moreover, such a division requires 20 × *N*_frag_ parameters, where *N*_frag_ is the number of fragments. This counteracts the purpose of introducing TLS parameters in the first place, which was to reduce the number of parameters.

The second factor that played a role in our successful TLS refinement is the fact that the molecule is conveniently situated on a centre of symmetry in our crystal structure of interest, further reducing the number of parameters from 20 to 12. Biologically active molecules, a large group of compounds that are currently actively investigated with X-ray powder diffraction, rarely possess molecular symmetry and are therefore rarely able to occupy special positions. A TLS refinement will be more complicated for this class of compounds, even if the rigid-body approximation is applicable.

### 3.6. Extension to other systems: MD simulations   

How transferable are the MD simulations presented here to other systems? The accuracy of force fields is limited, and there is no guarantee that starting an MD simulation with a force field reproduces phase transitions observed in experiments. As a case in point, initial calculations on 1,2,4,5-tetra­bromo­benzene showed that the crystal structures of the low-and the high-temperature phases, which both happen to be known for this jumping crystal and which (again) happen to be very similar, both converged to the structure of the high-temperature phase upon energy minimization at 0 K with the COMPASS force field. DFT-D recognizes the two structures as distinct minima, even at 0 K, so the neglect of thermal motion does not seem to explain this failure of the force field for the high-temperature phase; the fact that halogen atoms are notoriously anisotropic in the solid state and not described well by the spherical approximations in force fields, on the other hand, does. A more thorough comparison of the performance of four off-the-shelf force fields for the condensed phases of seven molecules in terms of the reproduction of unit-cell parameters and ADPs was published by Nemkevich *et al.* (2010 ▸), and their conclusions agree with ours that qualitative results are generally good, but quantitatively the results are off by significant margins. In fact, there is no guarantee that a molecular compound can be described by an off-the-shelf force field at all: the energy potential of the palladium-containing organometallic compound reported by Panda *et al.* (2014 ▸) to jump and shatter cannot be set up with the COMPASS force field. Our preference in this paper for a compound consisting solely of formally *sp* ^3^ carbon atoms and hydrogen atoms, which can both be described very well by the approximations inherent in the COMPASS force field, is therefore by no means a coincidence, but is dictated by the failure of the computational methods for alternative systems. A (partial) solution is the introduction of tailor-made force fields (TMFFs): force fields parameterized for one specific chemical compound at a time, allowing for more rigour in the description of the details of the energy potential of that compound (Neumann, 2008 ▸). In a previous paper, we described how MD with a tailor-made force field for cocaine describes the room-temperature crystal structure of cocaine very well (Li *et al.*, 2017 ▸). The accuracy of these force fields can be further improved by, for example, introducing anisotropic van der Waals interactions for halogens (Day *et al.*, 2005 ▸, see section 4.4.2) or replacing the atomic point charges by atomic multipoles (Pyzer-Knapp *et al.*, 2016 ▸).

The similarity of the structures before and after the phase transition suggests that the energy barrier between them is small, which in all likelihood also played a major role in the straightforward reproduction of the phase transition. Waiting for several seconds, minutes or longer until a phase transition has been initiated, as is possible in experiments, is not an option for atomistic simulations. Fortunately, in computational simulations, larger barriers may in principle be overcome by simply heating until the kinetic energy is sufficient to overcome the barrier within the short time scales of the simulation. Such an approach overshoots the actual transition temperature, possibly by a fair amount, but with a discrepancy of 85 K for this presumably best-case system the quantitative reproduction of the phase-transition temperature was never impressive.

Jumping crystals are not the only materials where the starting material and the experimental conditions leading to the final product are known, while the structure of the product is not: an obvious class of interesting materials that require a similar approach involving MD to investigate the structure of the resulting phase at the molecular level are ‘dehydrated hydrates’. In the pharmaceutical industry, sometimes initially the crystal structure of the solid form has water molecules incorporated into the crystal structures, forming a hydrate (Griesser, 2006 ▸). Further processing may remove this water, resulting in a dehydrated hydrate, the structure of which is not necessarily the same as that of any known anhydrate of the compound (*e.g*. obtained by crystal growth under the exclusion of water). Again, the hydrate crystals tend to disintegrate during the dehydration process, hampering the determination of the relevant phase at the molecular level. For these dehydrated hydrates a modified MD protocol, allowing the water molecules to be removed, would potentially enable full insight into the dehydration process and product at the molecular level (Larsen, Rantanen *et al.*, 2017 ▸; Larsen, Ruggiero *et al.*, 2017 ▸).

## 4. Conclusions   

When an experimental starting point and procedure are known, computer simulations are, in principle, able to take us to the destination, even if that destination cannot be characterized or even accessed experimentally, providing in the process, a wealth of molecular-level information that might have been difficult or impossible to obtain directly through experiment. Whilst a simple model system was chosen to demonstrate this proof-of-principle, we are confident that improvements in computational methods will make these simulations useful for more complex systems, such as the elucidation of the structures of dehydrated hydrates in the pharmaceutical industry, perhaps, when combined with crystal structure prediction, even before the compounds have been synthesized.

## 5. Software availability   

The in-house software used to analyse the MD trajectory and to generate the input for the TLS refinement with *TOPAS* was written in C++ and is available from the corresponding author upon request.

## Supplementary Material

Crystal structure: contains datablock(s) global, I. DOI: 10.1107/S205225251801686X/fc5028sup1.cif

Rietveld powder data: contains datablock(s) I. DOI: 10.1107/S205225251801686X/fc5028Isup2.rtv

Example TOPAS input file for TLS refinement.. DOI: 10.1107/S205225251801686X/fc5028sup3.txt

CCDC reference: 1867396

## Figures and Tables

**Table 1:** Selected parameters of Rietveld refinements with different models for the thermal ellipsoids

	Isotropic†	Scaled ADPs from MD‡	TLS§
*R*_wp_/*R*′_wp_ ¶	3.967/11.486	3.699/10.685	3.447/10.005
χ^2^	1.453	1.265	1.100
No. of fitted thermal parameters	1	1	12††
No. of thermal parameters describing ADPs of non-hydrogen atoms	1	48	48
Preferred orientation correction‡‡	0.950 (1)	0.973 (1)	0.986 (3)

† Fig. 6 ▸(*c*).

‡ Fig. 6 ▸(*a*).

§ Fig. 6 ▸(*b*).

¶ With and without background subtraction, respectively.

†† 20 if the molecule is not on a special position.

‡‡ March–Dollase correction with direction [010].

## Footnotes

1 In some space groups, unit cells with a 1 × 1 × 8 distribution of the molecules are possible, for example in *P*4_3_2_1_2, *Z*′ = 1 (see *e.g*. Beurskens *et al.*, 1980 ▸; CSD reference code MTKPIN) or a less exotic case, *P*2_1_2_1_2_1_, *Z*′ = 2 (see *e.g*. Birnbaum, 1977 ▸; CSD reference code HXUBIM10); *P*2_1_2_1_2_1_, *Z*′ = 2 is fairly common for biologically active compounds such as pharmaceuticals (Neumann & van de Streek, 2018 ▸). A 1 × 1 × 8 distribution is not commensurate with a 12 × 12 × 12 simulation box and hence, to capture all possible phase transitions, either a simulation box containing 24 × 24 × 24 = 13 824 molecules must be used, which would be prohibitively expensive in terms of the required hardware resources, or, as a more reasonable alternative, two separate simulations must be run, one with 12 × 12 × 12 = 1728 molecules and another with 8 × 8 × 8 = 512 molecules (or variations thereof, such as 16 × 8 × 8 = 1024 molecules or 16 × 16 × 8 = 2048 molecules to ensure that the simulation box is large enough in each direction to avoid self-interactions). It is not guaranteed however, that the initial phase allows the construction of such a simulation box. And even a 24 × 24 × 24 simulation box would not allow a phase transition to, for example, a *Z*′ = 5 phase, as 13 824 is not divisible by 5.
